# Hydrothermal vent fauna of the Galápagos Rift: updated species list with new records

**DOI:** 10.1007/s12526-024-01408-w

**Published:** 2024-02-15

**Authors:** Chong Chen, John W. Jamieson, Verena Tunnicliffe

**Affiliations:** 1 X-STAR, Japan Agency for Marine-Earth Science and Technology (JAMSTEC), 2-15 Natsushima-cho, Yokosuka, Kanagawa 237-0061 Japan; 2 Department of Earth Sciences, Memorial University of Newfoundland, St. John’s, Newfoundland A1B 3X5 Canada; 3 Department of Biology and School of Earth/Ocean Sciences, University of Victoria, Victoria, British Columbia V8P 3E6 Canada

**Keywords:** Biogeography, Chemosynthesis, Deep sea, Faunal list, Galápagos Spreading Center

## Abstract

The sighting of giant bivalves and tubeworms at the Rose Garden vent field on the Galápagos Rift in 1977 marked the discovery of hydrothermal vents, a turning point for modern biology. The following decade saw a flurry of taxonomic descriptions of vent endemic species from the first vents. With the finding of high-temperature “black smokers” on the East Pacific Rise, exploration shifted away from Galápagos. A faunal list of Galápagos vents with 65 species was published in 1991, then updated to 74 species in 2006. Since then, few expeditions returned to the Galápagos Rift. Here, we revisited several Galápagos vents including recently confirmed high-temperature sites and inactive sulfide mounds. From our collecting efforts and observations, as well as revisions from the literature, we update the faunal list to 92 species including 15 new records, restricted to obvious vent associates. Accurate regional faunal lists are important for understanding the biogeography of vent fauna, and our list will also be valuable for setting management strategies.

## Introduction

The discovery of hydrothermal vents themselves on the eastern Galápagos Rift in 1977 was not really a surprise, as geologists had predicted their presence from the missing heat measured near ridge axes (Sclater and Klitgord 1973) and warm buoyant plumes collected by a towed vehicle (Weiss et al. 1977). But nobody was prepared for the first contact with its bizarre inhabitants at a supposedly nutrient-deficient deep seabed two and a half kilometers below the surface—dense aggregations of giant clams and mussels, worms living in meter-long white tubes with red plumes swaying in shimmering water, and all other animals living with them (Corliss et al. 1979). Starting with the description of the giant vent clam *Turneroconcha magnifica* and the bythograeid vent crab *Bythograea thermydron* (Boss and Turner 1980; Williams 1980) followed by the giant tubeworm *Riftia pachyptila* and the discovery of chemosymbiosis (Cavanaugh et al. 1981; Jones 1981), biologists began to tease apart the taxonomic affinities and evolutionary origins of these creatures (Hessler and Smithey 1983).

Within a decade of its discovery, nearly all animals found in the original diffuse flow Galápagos Rift vents, now known as the Rose Garden vent field, were described. An early faunal list in 1991 included 65 species (Tunnicliffe 1991, 1992), and another in 2006 listed 74 (Desbruyères et al. 2006). With the discovery of “black smoker” chimneys spewing out high-temperature fluids in other systems such as East Pacific Rise (EPR) and Juan de Fuca Ridge (Tunnicliffe et al. 1985; Desbruyères and Laubier 1986), exploration shifted away from Galápagos Rift where vigorous venting was apparently lacking. An expedition in 2002 found that Rose Garden had been buried under fresh basaltic lava flows, and the communities had been largely wiped out except some recent settlers on nearby low-temperature venting from cracks in an area named Rosebud (Shank et al. 2003). The 2002 expedition also found signals for more vents east of Rose Garden on the eastern rift, plus a vent at the western Galápagos Rift near the Galápagos Islands. Between 2005 and 2006, towed-camera surveys in the western Galápagos Rift confirmed the first high-temperature chimneys (Haymon et al. 2008). Though these sites provide likely grounds for new records and subsequent research cruises have visited some of these areas using underwater vehicles (Shank et al. 2012; Raineault et al. 2016), no faunal updates have been published to date.

From October to November 2023, we were able to revisit the Galápagos Rift vents on-board the Schmidt Ocean Institute’s R/V *Falkor (too)* during the research cruise FKt231024. One aim of the cruise was to investigate the distributions of animal communities associated with both active and inactive vents. Here, we revise the faunal list of Galápagos Rift hydrothermal vents based on the literature since the last compilation (Desbruyères et al. 2006) plus new findings from our research expedition, in order to present all reliable distribution records from vents in this region.

## Materials and methods

**Fig. 1:**
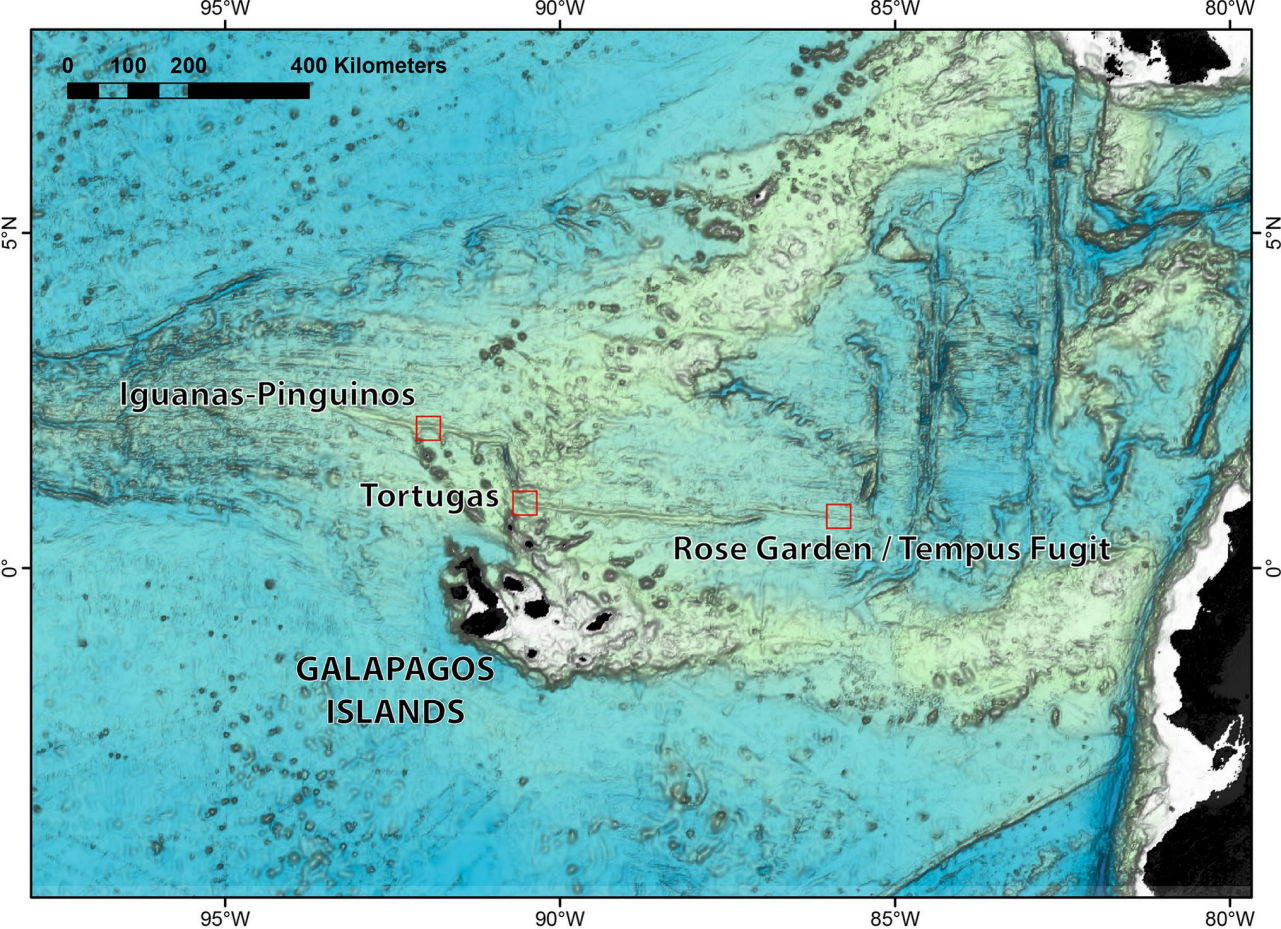
Map of the study area, showing the location of each hydrothermal vent field visited during R/V *Falkor (too)* research cruise Fkt231024

During R/V *Falkor (too)* cruise Fkt231024, we visited several hydrothermal vent fields on the Galápagos Rift using the remotely operated vehicle (ROV) *SuBastian*. These included Rose Garden/Rosebud (0.81° N, 86.22° W, 2450–2550 m deep; dive #603) and Tempus Fugit (0.77° N, 85.91–93° W, 2500–2560 m; dive #606–607, 609) on the eastern Galápagos Rift (Shank et al. 2012; Raineault et al. 2016); as well as Iguanas-Pinguinos (2.10° N, 91.89–94° W, 1650–1700 m; dive #611–613) and Tortugas, a newly-discovered active vent field on the western edge of the East Los Huellos Caldera (0.95° N, 90.53–56° W, 1500–1600 m; dive #614) on the western Galápagos Rift (Haymon et al. 2008). An overview map of the study area is presented in Fig. 1.

We used video and screengrabs from a 4K ultra-HD video camera (SULIS Subsea Z70; resolution 3840 × 2160 pixels) on the ROV *SuBastian* for seafloor imaging, which allowed up to 12 × zoom for close-up observations of even smaller animals. Animals were collected using either a seven-function manipulator arm (Schilling Robotics TITAN 4) or a suction sampler mounted on ROV *SuBastian*. Upon recovery on-board, animals were sorted in cold (4 °C) seawater, cleaned with a brush, and photographed using a Canon EOS 5Ds R digital single-lens reflex camera equipped with a Canon EF 100 mm F2.8L MACRO IS USM macro lens. Most new records are based on collected specimens, but some larger fauna were identified using close-up imagery. For peltospirid gastropods, which exhibited considerable morphological variability compared to specimens known from the EPR (McLean 1989a), the barcoding fragment of the mitochondrial cytochrome *c* oxidase subunit I (COI) gene was amplified and sequenced using the universal primer pairs HCO2198-LCO1490 (Folmer et al. 1994) following a published protocol (Chen et al. 2018) to confirm their identities. The new sequences were deposited on GenBank (PP000825-PP000827) and were compared with existing EPR sequences using the search function and the built-in pairwise distance calculator of NCBI BLAST.

Previously published faunal lists for the Galápagos Rift vents (Tunnicliffe 1991, 1992; Desbruyères et al. 2006) were examined for taxonomic status using both the World Register of Marine Species (WoRMS Editorial Board 2023) and primary literature. We aimed to remove erroneous records and to ensure the list only includes those species that rely strongly on the vent environment. To eliminate erroneous records, occurrence records at the Galápagos Rift were checked against the original descriptions and subsequent works on each species; geographic distribution of those species found in other hydrothermal systems were also recorded (see Table 1). New species described since the publication of the previous lists were checked in Google Scholar using search terms “Galapagos AND hydrothermal AND new species”. New records from our present study are added to this “historical” list.

**Table 1:** List of species recorded at Galápagos Rift hydrothermal vent systems. Species newly recorded in this study are listed in bold, species records from imagery only are denoted with asterisks and their species-level identification should be considered tentative. Abbreviations: GAL = Galápagos Rift; EPR = East Pacific Rise; GMS = Guaymas Basin; MBC = Monterey Bay, California; JdF = Juan de Fuca Ridge. “?” in distribution means occurrence outside Galápagos Rift remains uncertain

Phylum	Major clade	Order/family	Species	Distribution source	Distribution
Annelida	Hirudinea	Piscicolidae	*Bathybdella sawyeri* Burreson, 1981	(Burreson 1981)	GAL, EPR
Annelida	Polychaeta	Ampharetidae	*Amphisamytha galapagensis* Zottoli, 1983	(Zottoli 1983)	GAL, EPR, GMS
Annelida	Polychaeta	Alvinellidae	*Alvinella pompejana* Desbruyères & Laubier, 1986	(Raineault et al. 2016)	GAL, EPR
**Annelida**	**Polychaeta**	**Alvinellidae**	***Alvinella caudata*** **Desbruyères & Laubier, 1986**	**This study**	GAL, EPR
Annelida	Polychaeta	Alvinellidae	*Paralvinella grasslei* Desbruyères & Laubier, 1982	(Desbruyères and Laubier 1982)	GAL, EPR
Annelida	Polychaeta	Amphinomidae	*Archinome rosacea* (Blake, 1985)	(Blake 1985; Borda et al. 2013)	GAL, EPR
Annelida	Polychaeta	Dorvilleidae	*Ophryotrocha akessoni* Blake, 1985	(Blake 1985)	GAL, EPR
**Annelida**	**Polychaeta**	**Eunicidae**	***Eunice*** **cf.** ***pulvinopalpata*** Fauchald, 1982*	**This study**	GAL, EPR
**Annelida**	**Polychaeta**	**Hesionidae**	***Hesiolyra bergi*** **Blake, 1985**	**This study**	GAL, EPR
Annelida	Polychaeta	Hesionidae	*Hesiospina vestimentifera* Blake, 1985	(Blake 1985)	GAL, EPR
Annelida	Polychaeta	Hesionidae	*Nereimyra alvinae* Blake, 1985 (*nomen dubium*)	(Blake 1985; Pleijel et al. 2012)	GAL, GMS
Annelida	Polychaeta	Maldanidae	*Nicomache arwidssoni* Blake, 1985	(Blake 1985)	GAL, EPR
Annelida	Polychaeta	Nereididae	*Nereis sandersi* Blake, 1985	(Blake 1985)	GAL, EPR
Annelida	Polychaeta	Phyllodocidae	*Galapagomystides aristata* Blake, 1985	(Blake 1985)	GAL, EPR
Annelida	Polychaeta	Polynoidae	*Branchinotogluma hessleri* Pettibone, 1985	(Pettibone 1985a)	GAL, EPR
Annelida	Polychaeta	Polynoidae	*Branchinotogluma sandersi* Pettibone, 1985	(Pettibone 1985a)	GAL, EPR
Annelida	Polychaeta	Polynoidae	*Branchipolynoe symmytilida* Pettibone, 1984	(Pettibone 1984a)	GAL, EPR
Annelida	Polychaeta	Polynoidae	*Harmothoe macnabi* Pettibone, 1985	(Pettibone 1985b, 1990)	GAL. JdF
Annelida	Polychaeta	Polynoidae	*Lepidonotopodium riftense* Pettibone, 1984	(Pettibone 1984b)	GAL, EPR
Annelida	Polychaeta	Polynoidae	*Lepidonotopodium williamsae* Pettibone, 1984	(Pettibone 1984b)	GAL, EPR
Annelida	Polychaeta	Polynoidae	*Levensteiniella kincaidi* Pettibone, 1985	(Pettibone 1985b)	GAL, EPR
Annelida	Polychaeta	Polynoidae	*Macellicephala galapagensis* Pettibone, 1985	(Pettibone 1985b)	GAL
Annelida	Polychaeta	Serpulidae	*Laminatubus alvini* Ten Hove & Zibrowius, 1986	(Ten Hove and Zibrowius 1986)	GAL, EPR
Annelida	Polychaeta	Serpulidae	*Protis hydrothermica* Ten Hove & Zibrowius, 1986	(Ten Hove and Zibrowius 1986)	GAL, EPR
Annelida	Polychaeta	Siboglinidae	*Riftia pachyptila* Jones, 1985	(Jones 1981)	GAL, EPR
**Annelida**	**Polychaeta**	**Siboglinidae**	*Oasisia alvinae* Jones, 1985*	**This study**	GAL, EPR
**Annelida**	**Polychaeta**	**Siboglinidae**	***Tevnia jerichonana* Jones, 1985**	**This study**	GAL, EPR
Annelida	Polychaeta	Spionidae	*Laubieriellus grasslei* Maciolek, 1981	(Maciolek 1981)	GAL
Annelida	Polychaeta	Spionidae	*Prionospio sandersi* Maciolek, 1981	(Maciolek 1981)	GAL, EPR
Annelida	Polychaeta	Spionidae	*Xandaros acanthodes* Maciolek, 1981	(Maciolek 1981)	GAL
Arthropoda	Anomura	Munidopsidae	*Munidopsis recta* Baba, 2005	(Jones and Macpherson 2007)	GAL, EPR
Arthropoda	Acari	Halacaridae	*Copidognathus papillatus*	(Krantz 1982)	GAL, EPR, JdF
**Arthropoda**	**Brachyura**	**Bythograeidae**	*Cyanagraea praedator* de Saint Laurent, 1984*	**This study**	GAL, EPR
Arthropoda	Brachyura	Bythograeidae	*Bythograea galapagensis* Guinot & Hurtado, 2003	(Guinot and Hurtado 2003)	GAL
Arthropoda	Brachyura	Bythograeidae	*Bythograea intermedia* de Saint Laurent, 1988 (*nomen dubium*)	(de Saint Laurent 1988)	GAL
Arthropoda	Brachyura	Bythograeidae	*Bythograea thermydron* Williams, 1980	(Williams 1980)	GAL, EPR
Arthropoda	Caridea	Alvinocarididae	*Alvinocaris lusca* Williams & Chace, 1982	(Williams and Chace 1982)	GAL, EPR
**Arthropoda**	**Caridea**	**Thoridae**	*Lebbeus laurentae* Wicksten, 2010*	**This study**	GAL, EPR
Arthropoda	Copepoda	Harpacticoida	*Argestoides prehensilis* Huys & Conroy-Dalton, 1987	(Humes and Segonzac 1998)	GAL
Arthropoda	Copepoda	Harpacticoida	*Andromastax muricatus* Conroy-Dalton & Huys, 1999	(Conroy-Dalton and Huys 1999)	GAL
Arthropoda	Copepoda	Poecilostomatoida	*Oncaea praeclara* Humes, 1988	(Humes 1988a)	GAL, EPR
Arthropoda	Copepoda	Siphonostomatoida	*Aphotopontius arcuatus* Humes, 1987	(Humes 1987)	GAL, EPR
Arthropoda	Copepoda	Siphonostomatoida	*Aphotopontius baculigerus* Humes, 1987	(Humes 1987)	GAL
Arthropoda	Copepoda	Siphonostomatoida	*Aphotopontius limatulus* Humes, 1987	(Humes 1987)	GAL, EPR
Arthropoda	Copepoda	Siphonostomatoida	*Aphotopontius mammillatus* Humes, 1987	(Humes 1987)	GAL, EPR
Arthropoda	Copepoda	Siphonostomatoida	*Aphotopontius probolus* Humes, 1990	(Humes 1990)	GAL
Arthropoda	Copepoda	Siphonostomatoida	*Ceuthoecetes acanthothrix* Humes, 1987	(Humes 1987)	GAL, EPR
Arthropoda	Copepoda	Siphonostomatoida	*Ceuthoecetes aliger* Humes & Dojiri, 1980	(Humes and Dojiri 1980)	GAL, EPR
Arthropoda	Copepoda	Siphonostomatoida	*Ceuthoecetes cristatus* Humes, 1987	(Humes 1987)	GAL, EPR
Arthropoda	Copepoda	Siphonostomatoida	*Ceuthoecetes introversus* Humes, 1987	(Humes 1987)	GAL, EPR
Arthropoda	Copepoda	Siphonostomatoida	*Ecbathyrion prolixicauda* Humes, 1987	(Humes 1987)	GAL, EPR
Arthropoda	Copepoda	Siphonostomatoida	*Hyalopontius boxshalli* Humes, 1988	(Humes 1988b)	GAL
Arthropoda	Copepoda	Siphonostomatoida	*Nilva torifera* Humes, 1987	(Humes 1987)	GAL, EPR
Arthropoda	Copepoda	Siphonostomatoida	*Rhogobius contractus* Humes, 1987	(Humes 1987)	GAL, EPR
Arthropoda	Copepoda	Siphonostomatoida	*Rhogobius pressulus* Humes, 1989	(Humes 1989)	GAL
**Arthropoda**	**Cirripedia**	**Balanomorpha**	***Eochionelasmus*** cf.* paquensis *Yamaguchi & Newman, 1997*	**This study**	GAL, ?
Arthropoda	Leptostraca	Nebaliidae	*Dahlella caldariensis* Hessler, 1984	(Hessler 1984)	GAL, EPR
Arthropoda	Peracarida	Amphipoda	*Apotectonia heterostegos* Barnard & Ingram, 1990	(Barnard and Ingram 1990)	GAL
Arthropoda	Peracarida	Amphipoda	*Hirondellea glutonis* Barnard & Ingram, 1990	(Barnard and Ingram 1990)	GAL, EPR
Arthropoda	Peracarida	Amphipoda	*Stephonyx mytilus* (Barnard & Ingram, 1990)	(Barnard and Ingram 1990)	GAL, EPR
Arthropoda	Peracarida	Amphipoda	*Ventiella sulfuris* Barnard & Ingram, 1990	(Barnard and Ingram 1990)	GAL, EPR
**Arthropoda**	**Pycnogonida**	**Ammotheidae**	***Sericosura cyrtoma* Child & Segonzac, 1996**	**This study**	**GAL, EPR**
**Arthropoda**	**Pycnogonida**	**Ammotheidae**	***Sericosura*** **sp.**	**This study**	GAL, ?
**Cnidaria**	**Hydrozoa**	**Candelabridae**	***Candelabrum*** **cf.** ***phrygium* (Fabricius, 1780)**	**This study**	GAL, ?
Cnidaria	Hydrozoa	Rhodaliidae	*Thermopalia taraxaca* Pugh, 1983	(Pugh and Marshall 1983)	GAL, EPR
Mollusca	Bivalvia	Mytilidae	*Bathymodiolus thermophilus* Kenk & Wilson, 1985	(Kenk and Wilson 1985)	GAL, EPR
Mollusca	Bivalvia	Propeamussiidae	*Catillopecten vulcani* (Schein-Fatton, 1985)	(Schein-Fatton 1985)	GAL, EPR
Mollusca	Bivalvia	Vesicomyidae	*Turneroconcha magnifica* (Boss & Turner, 1980)	(Boss and Turner 1980)	GAL, EPR
Mollusca	Gastropoda	Lepetodrilidae	*Clypeosectus delectus* McLean, 1989	(McLean 1989b)	GAL, EPR
Mollusca	Gastropoda	Lepetodrilidae	*Gorgoleptis patulus* McLean, 1988	(McLean 1988)	GAL, EPR
Mollusca	Gastropoda	Lepetodrilidae	*Lepetodrilus cristatus* McLean, 1988	(McLean 1988)	GAL, EPR
Mollusca	Gastropoda	Lepetodrilidae	*Lepetodrilus elevatus galriftensis* McLean, 1988	(McLean 1988; Matabos and Jollivet 2019)	GAL, EPR
Mollusca	Gastropoda	Lepetodrilidae	*Lepetodrilus ovalis* McLean, 1988	(Tyler et al. 2008)	GAL, EPR, MBC
Mollusca	Gastropoda	Lepetodrilidae	*Lepetodrilus pustulosus* McLean, 1988	(McLean 1988)	GAL, EPR
Mollusca	Gastropoda	Lepetodrilidae	*Lepetodrilus* aff. *tevnianus* McLean, 1993	(Matabos and Jollivet 2019)	GAL
Mollusca	Gastropoda	Neomphalidae	*Lacunoides exquisitus* Warén & Bouchet, 1989	(Warén and Bouchet 1989)	GAL
Mollusca	Gastropoda	Neomphalidae	*Neomphalus fretterae* McLean, 1981	(McLean 1981)	GAL, EPR
Mollusca	Gastropoda	Melanodrymiidae	*Melanodrymia *sp.	(Gustafson 1991; Warén and Bouchet 1993)	GAL, ?
Mollusca	Gastropoda	Neolepetopsidae	*Eulepetopsis vitrea* McLean, 1990	(McLean 1990)	GAL, EPR
Mollusca	Gastropoda	Neolepetopsidae	*Neolepetopsis densata* McLean, 1990	(Gustafson and Lutz 1994)	GAL, EPR
**Mollusca**	**Gastropoda**	**Peltospiridae**	***Nodopelta heminoda*** **McLean, 1989**	**This study**	GAL, EPR
**Mollusca**	**Gastropoda**	**Peltospiridae**	***Peltospira delicata*** **McLean, 1989**	**This study**	GAL, EPR
**Mollusca**	**Gastropoda**	**Peltospiridae**	***Peltospira operculata*** **McLean, 1989**	**This study**	GAL, EPR
Mollusca	Gastropoda	Provannidae	*Provanna ios* Warén & Bouchet, 1986	(Warén and Bouchet 1986)	GAL, EPR
Mollusca	Gastropoda	Provannidae	*Provanna muricata* Warén & Bouchet, 1986	(Warén and Bouchet 1986)	GAL, EPR
Mollusca	Gastropoda	Raphitomidae	*Nepotilla *sp.	(Gustafson 1991; Warén and Bouchet 1993)	GAL, ?
**Mollusca**	**Gastropoda**	**Raphitomidae**	***Phymorhynchus major* Warén & Bouchet, 2001**	**This study**	GAL, EPR
Mollusca	Solenogastres	Simrothiellidae	*Helicoradomenia acredema* Scheltema, 2000	(Scheltema 2000)	GAL, EPR
Hemichordata	Enteropneusta	Harrimaniidae	*Saxipendium coronatum* Woodwick & Sensenbaugh, 1985	(Woodwick and Sensenbaugh 1985)	GAL, EPR
Chordata	Teleostei	Bythitidae	*Thermichthys hollisi* (Cohen, Rosenblatt & Moser, 1990)	(Cohen et al. 1990)	GAL, EPR
Chordata	Teleostei	Zoarcidae	*Pachycara rimae* Anderson, 1989	(Anderson 1989)	GAL
Chordata	Teleostei	Zoarcidae	*Thermarces cerberus* Rosenblatt & Cohen, 1986	(Rosenblatt and Cohen 1986)	GAL, EPR

## Results and discussion

### Overview of vent fields visited

In the eastern Galápagos Rift, the Rose Garden/Rosebud area was covered by fresh basaltic lava flow and devoid of living vent fauna. This confirms the finding from a 2011 cruise that another eruption event between 2005 and 2011 had eliminated fauna in this field (Shank et al. 2012). Furthermore, we revisited a serpulid worm colony found in 2015 (Raineault et al. 2016) in case living vent fauna persisted (0.8049° N, 86.2194° W, 2447 m deep), but only found decaying serpulid tubes and dissolving mussel shell debris. As such, venting at Rose Garden has likely ceased—although we did not visit the location of the East of Eden field. In the nearby Tempus Fugit field (Raineault et al. 2016), we found that venting at the previously known main diffuse flow site (0.7700° N, 85.9114° W, 2561 m deep) had waned, with few living vesicomyid clams and *Riftia* tubeworms. Nevertheless, we found a new diffuse flow vent nearby (0.7712° N, 85.9236° W, 2602 m deep; “Walking Dead” vent). We also revisited the active chimney (Raineault et al. 2016) at the western end of Tempus Fugit (0.7712° N, 85.9332° W, 2514 m deep; “Zombie” vent) and confirmed high-temperature (>200 °C; measured with the ROV temperature probe) venting there. A number of dead spires or inactive mounds were found around the Zombie vent and were also surveyed.

Shifting to the western Galápagos Rift, we revisited all three vent sites in the Iguanas-Pinguinos vent field (Haymon et al. 2008; Raineault et al. 2016), including Iguanas East (2.0992° N, 91.9053° W, 1670 m), Iguanas West (2.1050° N, 91.9378° W, 1670 m), and Pinguinos (2.0993° N, 91.9052° W, 1670 m). We confirmed chimney structures associated with vigorous venting of high-temperature fluid at all three locations. At East Los Huellos Caldera, where only plume signals were known (Haymon et al. 2008), we discovered active venting associated with chemosynthetic communities. This included both diffuse venting areas dominated by mussels and vesicomyid clams (0.9546° N, 90.5566° W, 1590 m) and active chimney complexes with high-temperature (>250° C) venting (0.9543° N, 90.5613°W, 1520 m).

### Revising the existing faunal list

The faunal list of the Galápagos Rift vents in Desbruyères et al. (2006) included a total of 74 species. Of these, two orbiniid annelid species including *Orbiniella aciculata* and *Scoloplos ehlersi* were erroneously included in the list, as the author clearly states these were collected from box cores deployed near the Galápagos Rift but were not from the vent community (Blake 1985). Here, we further remove the lysianassoid amphipod *Abyssorchomene abyssorum* on the grounds that it is a globally distributed deep-sea species found in non-chemosynthetic seafloor and that its vent record is based on a single specimen that may have been a by-catch (Barnard and Ingram 1990). Similarly, we took out the abyssal grenadier *Coryphaenoides armatus* since it is merely an occasional visitor to vents from the surrounding deep sea. Though there are two species of dubious taxonomic status—the hesionid polychaete *Nereimyra alvinae* with poorly preserved types (Pleijel et al. 2012) and the crab *Bythograea intermedia* described from megalopa and juveniles only (de Saint Laurent 1988)—we have kept them, pending future taxonomic revision. The melanodrymid snail *Melanodrymia* sp. and the raphitomid snail *Nepotilla* sp. were initially reported in a conference abstract (Gustafson 1991) and then included in a gastropod faunal list by taxonomic experts (Warén and Bouchet 1993). Though their species-level identification remains unclear, they remain on the list pending more taxonomic information. Moalic et al. (2012) supplemented the list in Desbruyères et al. (2006) to report 83 taxa. In addition to the annelids above, occasional visitors, and a double record of *Thermichthys hollisi*, we removed species we could not verify such as polychaetes only known from Juan de Fuca/Gorda Ridges, *Bythograea microps*, and *Aphotopontius acanthinus*. There remained 74 species.

Since the 2006 list was published, three additional species have been recorded from Galápagos Rift in the published literature. The first is the squat lobster *Munidopsis recta* Baba, 2005, that was confirmed as a Galápagos record by Jones and Macpherson (2007) using COI sequencing. The second species is the Pompeii worm *Alvinella pompejana*, visually confirmed from Tempus Fugit vent field in 2010, but not sampled (Raineault et al. 2016). The third species is *Lepetodrilus* aff. *tevnianus* Galápagos sensu Matabos and Jollivet (2019), morphologically resembling *Lepetodrilus tevnianus* found on the EPR vents but is a genetically distinct lineage considered to represent an undescribed species (Matabos and Jollivet 2019). Altogether, these bring the historical species occurrence record to 77 species.

### New records

**Fig. 2:**
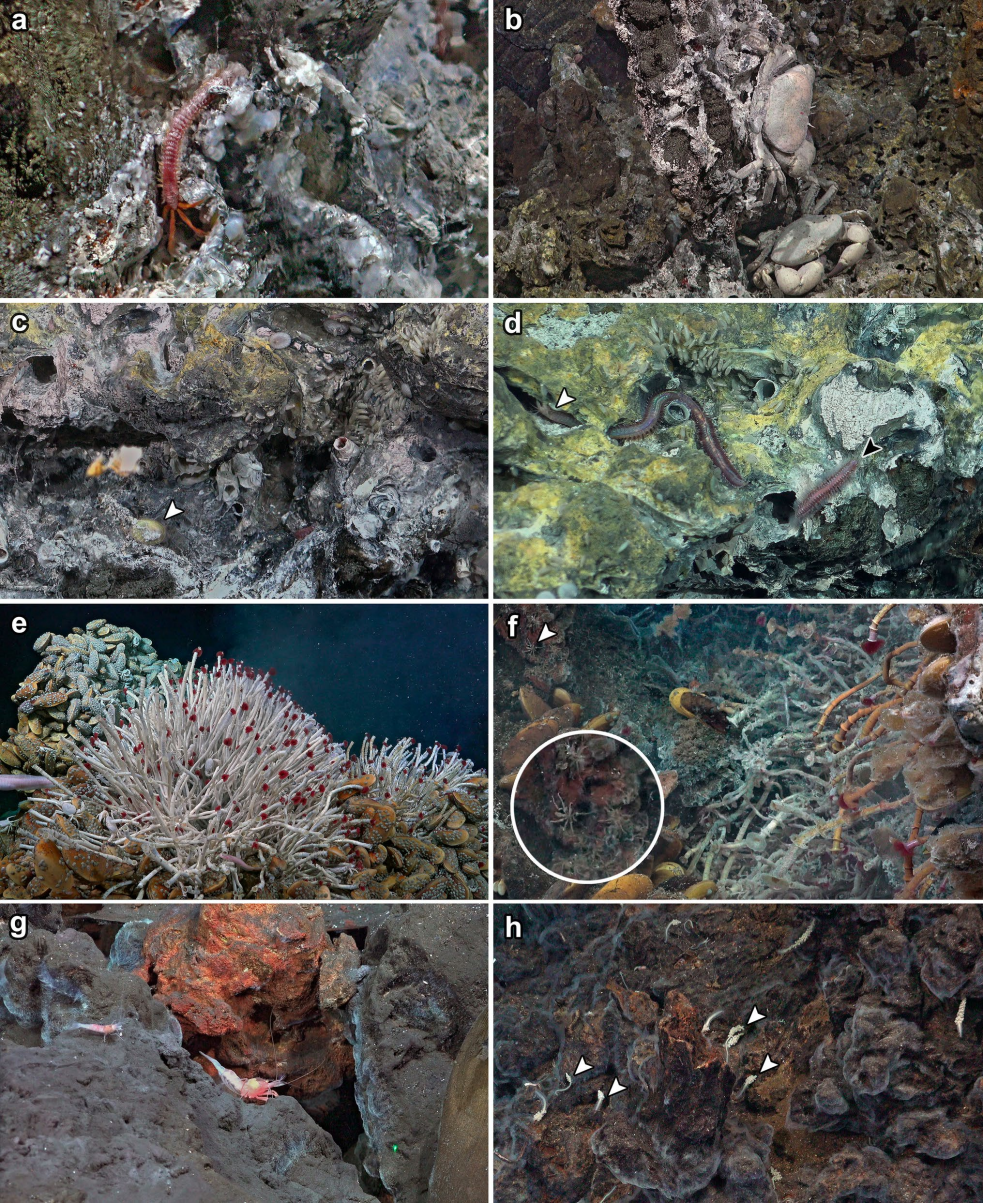
In situ imagery of Galápagos Rift vents captured by screengrabs of the 4K video camera in the present study: **a** the alvinellid worm *Alvinella caudata* on chimney wall of Zombie vent, Tempus Fugit; **b** two individuals of the bythograeid crab *Cyanagraea praedator*, Zombie vent, Tempus Fugit; **c** a living individual of *Nodopelta heminoda* (white arrow), Zombie vent, Tempus Fugit; **d** the polychaete worms *Eunice* cf. *pulvinopalpata* (white arrow) and *Hesiolyra bergi* (black arrow), Zombie vent, Tempus Fugit; **e** a bouquet of *Tevnia jerichonana* tubeworms at a peripheral diffuse flow at West Iguanas, Iguanas-Pinguinos; **f** a cluster of *Oasisia alvinae* tubeworms at a diffuse flow site in East Los Huellos Caldera and several individuals of *Sericosura* pycnogonids nearby (white arrow and enlarged in inset; likely a mix of both species in Table 1); **g** *Lebbeus laurentae* (larger shrimp on the right) seen with *Alvinocaris lusca* at the base of the active chimney complex at West Iguanas, Iguanas-Pinguinos; **h** several hydrozoan *Candelabrum* cf. *phrygium* near a low-temperature vent at West Iguanas, Iguanas-Pinguinos (representative individuals indicated by white arrows)

**Fig. 3:**
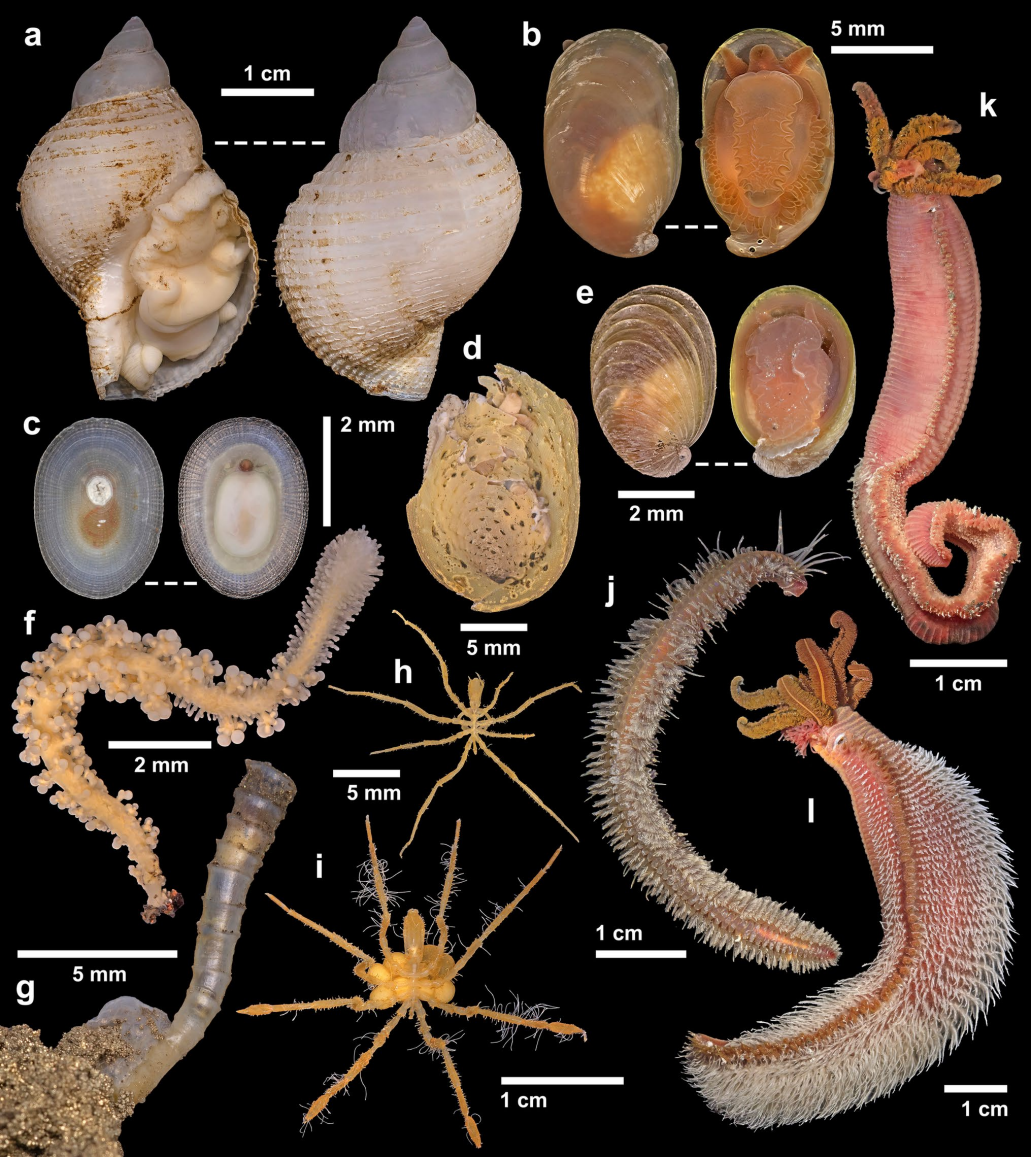
Specimens collected from Galápagos Rift vents in the present study. **a** *Phymorhynchus major*, Walking Dead diffuse flow vent, Tempus Fugit; **b** *Peltospira* *delicata*, Zombie vent, Tempus Fugit; **c** *Neolepetopsis densata*, inactive spires near Zombie vent, Tempus Fugit; **d** *Nodopelta heminoda*, Zombie vent, Tempus Fugit; **e** young individual of *Peltospira operculata*, Zombie vent, Tempus Fugit; **f** *Candelabrum* cf. *phrygium*, West Iguanas, Iguanas-Pinguinos; **g** a juvenile individual of *Tevnia jerichonana* from Zombie vent, Tempus Fugit; **h** *Sericosura* sp., diffuse flow at East Los Huellos Caldera; **i** *Sericosura cyrtoma*, diffuse flow at East Los Huellos Caldera; **j** *Hesiolyra bergi*, Zombie vent, Tempus Fugit; **k** *Alvinella caudata*, Zombie vent, Tempus Fugit; **l** *Alvinella pompejana*, Zombie vent, Tempus Fugit

From our observations and collections during the 2023 cruise, we encountered a total of 15 species that are clearly associated with the chemosynthetic ecosystem and not previously recorded from Galápagos Rift vents. Table 1 lists our updated full faunal list comprising 92 species, with our new records shown in bold. Figure 2 presents key in situ screengrabs including records based on species clearly identifiable from imagery, while Fig. 3 shows photographs of specimens collected. In the following paragraphs, we provide more details on our newly recorded species.

The alvinellid worm *Alvinella caudata* (Figs. 2a and 3k) was seen on active chimney walls at Tempus Fugit, Iguanas-Pinguinos, and Tortugas. It co-occurred with *A. pompejana*, and both were collected together at Tempus Fugit; we show a specimen photo of *A. pompejana* (Fig. 3l) since this is the first time a specimen was collected from Galápagos Rift and serves as a confirmation of the previous record (Raineault et al. 2016). Also found on the same habitat was the hesionid worm *Hesiolyra bergi* (Figs. 2d and 3j), which occurred in aggregations on the chimneys; individuals were sometimes seen going into tubes of *Alvinella* worms. We give a tentative identification of *Eunice* cf. *pulvinopalpata* (Fauchald 1982) to a eunicid worm seen in the same habitat, but not collected (Fig. 2d). The bythograeid crab *Cyanagraea praedator* (Fig. 2b) was common on the active chimneys too, readily identified from images by the well-developed eye-stalk sockets and their large size (de Saint Laurent 1984). The association between *Cyanagraea* and *Alvinella* is also known from EPR vents, where the former is a predator of the latter (Desbruyères et al. 2006).

We also collected three species of peltospirid gastropods from the high-temperature Zombie vent at Tempus Fugit, including *Nodopelta heminoda* (Fig. 3d), *Peltospira delicata* (Fig. 3b), and *Peltospira operculata* (Fig. 3e). Although only one damaged specimen of *N. heminoda* could be collected, several individuals were seen near *Alvinella* tubes (Fig. 2c); the COI sequence of the collected specimen (GenBank PP000825) matched an existing mitogenome of the same species (GenBank BioProject PRJNA927338) with a pairwise identity of 99.82%. *Peltospira operculata* is also recorded based on a single young specimen (Fig. 3e) still displaying strongly ribbed shell sculpture that fades in adults (McLean 1989a). The spacing of its ribbing is wider than typical specimens from the EPR (McLean 1989a) but its COI sequence (GenBank PP000826) was closely comparable to five existing sequences (GenBank GU984275-GU984279) with pairwise identities between 99.19% and 99.68%, indicating this spacing is intraspecific variation. *Peltospira delicata*, recorded based on two collected adult specimens, was unusual in lacking clear spiral ridges on the body whorl (McLean 1989a). The COI sequence of the ethanol-preserved specimen (GenBank PP000827) matched an existing sequence of *P. delicata* (GenBank AY923931) with a pairwise identity of 99.85%. Though having a smooth adult shell is reminiscent of *Peltospira operculata*, all other external morphological features of our specimens such as the overall weaker coiling and the lack of operculum agree with identification as *P. delicata* (McLean 1989a; Warén and Bouchet 2001). This is indicative of a wider range of phenotypic variability in this species than previously known. Peltospirid snails were not seen on active chimneys in the western Galápagos Rift, but as we did not sample those chimneys for animals, we may have missed them on video due to their small size.

At both diffuse flow areas and active chimney walls in Iguanas-Pinguinos and Tortugas, we saw bouquets of the tubeworm *Tevnia jerichonana* (Fig. 2e), and a single juvenile specimen (Fig. 3g) was collected from the Zombie vent at Tempus Fugit where no adults could be seen. Only at the diffuse flow site at Tortugas did we see a cluster of *Oasisia alvinae*. The only tubeworm known from previous explorations in the eastern Galápagos Rift was *Riftia pachyptila* (Corliss et al. 1979; Jones 1981; Raineault et al. 2016), which also occurred in both diffuse flow sites in Tempus Fugit (but in lower abundance than previous expeditions due to waning activity there). Conversely, at the western Galápagos Rift, we did not see any sign of *Riftia*. At Tortugas, we found two species of the pycnogonid genus *Sericosura* in abundance around diffuse flows (Fig. 2f). One species with seven-segmented palps was readily identifiable as *Sericosura cyrtoma* (Fig. 3h), but the other (Fig. 3i) with nine-segmented palps did not match any described eastern Pacific congeners (Child 1987; Child and Segonzac 1996; Wang et al. 2013) and may represent an undescribed species. Though we did not find pycnogonids in the eastern Galápagos Rift, a previous cruise reported seeing pycnogonids there (Raineault et al. 2016), likely also *Sericosura*.

The raphitomid snail *Phymorhynchus* was often seen in the periphery zone of all vent fields we visited. Initially, *Phymorhynchus* from the Galápagos Rift was considered to be conspecific with those on the EPR (Warén and Bouchet 1989), but this distribution record was not mentioned when *P. major* was formally named based on only EPR material (Warén and Bouchet 2001). Here, we collected a specimen (Fig. 3a) and confirm the presence of *P. major* in the Galápagos. Though not seen on our expedition, we note that a recent expedition also on R/V *Falkor (too)* (Fkt230812) encountered dense coverage of a vent barnacle tentatively identified as *Eochionelasmus* cf. *paquensis* (Hiromi K. Watanabe, pers. comm.) at a vent site named Sendero del Cangrejo (2.53° N, 94.33° W, 2490 m deep). This species is added to our list based on imagery shown on an openly available YouTube stream of ROV *SuBastian* dive #573 at this site (Schmidt Ocean Institute 2023).

We also saw several individuals of *Lebbeus* co-occurring with *Alvinocaris lusca* on the vent periphery only in the West Iguanas vent (Fig. 2g). Though *Lebbeus* was not collected, our imagery provided sufficient resolution for its tentative identification as *L. laurentae* based on external morphology (Komai et al. 2012). Numerous individuals of the hydrozoan *Candelabrum* were seen also near the periphery of West Iguanas (Fig. 2h). The collected individual (Fig. 3f) was morphologically similar to *Candelabrum phrygium* which has a pan-arctic distribution and also known from Mid-Atlantic Ridge vents (Segonzac and Vervoort 1995). As Galápagos is far from its known range, we consider it likely to be a distinct species and tentatively identified it as *C.* cf. *phrygium*. Further away from high-temperature venting, we found many individuals of the true limpet *Neolepetopsis densata* on inactive chimneys near Zombie vent near Tempus Fugit (Fig. 3c). Although Gustafson and Lutz (1994) published a record for *N. densata* from an inactive mound on the Galápagos Rift, the validity of this was questionable as the figure captions listed the illustrated specimens as from Galápagos but the same figures were cited in the main text as specimens from 9 to 10° N on the EPR. Our present finding serves to confirm their Galápagos record. To our knowledge, this is the only Galápagos Rift vent-specific species likely restricted to inactive chimneys, a distribution pattern typical for genus *Neolepetopsis* (McLean 1990; Chen et al. 2021).

During our exploration, we also saw a number of animals typical of non-chemosynthetic seafloor environments within proximity to vents, such as the Pacific white skate *Bathyraja spinosissima* known to incubate egg cases at Galápagos Rift vents (Salinas-de-León et al. 2018), the octopus *Graneledone* (likely an undescribed species, Janet Voight pers. comm.) (Desbruyères et al. 2006), and some encrusting demosponges. We did not include them in our list due to the likely incidental nature of their presence in or near the chemosynthetic ecosystem.

We note that a limitation of our study is that some new records such as the tubeworm *Oasisia alvinae* or the vent crab *Cyanagraea praedator* were not collected and identified based on imagery data only, precluding future genetic studies. Though these species are easily identified from external morphology based on our current understanding with just one species in their respective genera in the eastern Pacific vents, we cannot rule out the presence of cryptic species specific to the Galápagos Rift. Previous studies in annelids and gastropods have highlighted the presence of genetic barriers and population subdivisions between the Galápagos Rift and the EPR (Hurtado et al. 2004; Matabos and Jollivet 2019). This includes cryptic species that are separated across the two ridge systems; for example, the limpet *Lepetodrilus elevatus* is known to consist of at least four cryptic genetic lineages across the eastern Pacific vents that are tentatively treated as one species (Matabos and Jollivet 2019).

Forty-five years after the discoveries at Rose Garden, there still are vent communities within 35 km of the original site. As the most easterly extension of the east/southeast Pacific biogeographic region, these vents may be both a population sink and a source of novel genetic diversity. Given the location in international waters near the large Galápagos mound sulfide deposits, consideration of protections such as Ecologically and Biologically Sensitive Area (EBSA) designation is warranted. The abundant active and inactive chimneys of the Iguanas-Pinguinos and Tortugas sites are testament to long-term hydrothermalism that has supported vent communities and diversification of the fauna. Currently, at least 14 species (not including *nomen dubium*) are known only from the Galápagos Rift (Table 1), an endemism proportion of 15% and another five species whose endemism is uncertain. While not high compared to the endemism among western Pacific vent systems (Tunnicliffe et al. 2023), as there are no geographic barriers separating the Rift from EPR, specific environmental conditions may foster the endemics. For example, sustained venting over numerous large chimneys may foster population maintenance compared to the high turnover at EPR vents (Gollner et al. 2017). Further collecting and molecular work is needed to investigate the biogeographic relationships between Galápagos Rift and the EPR in finer detail. Accurate species lists and occurrence data can reveal key processes driving the biogeographic patterns and evolution of hydrothermal vent fauna in general (Giguère and Tunnicliffe 2021; Brunner et al. 2022).

## Conclusions

We revised the existing faunal list of Galápagos Rift vents and added 15 new records based on our observations and specimens collected, bringing the total to 92 species. Of these species, 14 are only known from Galápagos Rift. Though only based on qualitative observations, our results suggest some differences in fauna composition of vents at eastern vs western Galápagos Rift, warranting future research. Diversity data provide important grounds for constructing management strategies and spatial planning, especially with the growing interests for deep-sea mineral resources. As the Galápagos Rift is partially included in the Galápagos Marine Reserve, our updated species list will also be useful for conservation and marine spatial planning in this world heritage site. On the one hand, we increase considerably the number of vent species (especially those living on high-temperature chimneys) living within the Reserve, including those lacking any formal protection on the extensive East Pacific Rise south of Mexican waters. On the other hand, we show that Galápagos Rift vents host several endemic species. This is further supplemented by cryptic lineages and genetic diversities not found outside Galápagos due to isolation from the EPR at least for some gastropods (Matabos and Jollivet 2019), likely also true for some newly recorded species herein. Altogether, our results highlight the Galápagos vents as a candidate for focused conservation efforts.

## Declarations

### Sampling and field studies

All necessary permits for sampling and observation have been obtained by the authors where applicable. Permission for ROV dives inside the Galápagos Marine Reserve in Ecuador was granted through a partnership between Schmidt Ocean Institute and the Charles Darwin Foundation (MAATE-DPNG/DGA-2023-1449-O).

### Data availability

Data relevant to our conclusions are included in the main text. Specimens collected within the Galápagos Marine Reserve are vouchered in the Charles Darwin Research Station on Santa Cruz Island (https://www.darwinfoundation.org/en/about/cdrs). All ROV SuBastian dives during cruise FKt231024 are available online through the Schmidt Ocean Institute YouTube page: https://www.youtube.com/@SchmidtOcean/streams.
